# Diagnosis of in vivo vertical root fracture using deep learning on cone-beam CT images

**DOI:** 10.1186/s12903-022-02422-9

**Published:** 2022-09-05

**Authors:** Ziyang Hu, Dantong Cao, Yanni Hu, Baixin Wang, Yifan Zhang, Rong Tang, Jia Zhuang, Antian Gao, Ying Chen, Zitong Lin

**Affiliations:** 1 Department of Dentomaxillofacial Radiology, Nanjing Stomatological Hospital, Medical School of Nanjing University, Zhong Yang Road 30, Nanjing City, 210008 Jiangsu People’s Republic of China; 2 School of Electronic Science and Engineering, Nanjing University, Nanjing, China; 3 Department of Stomatology, Guangdong Medical University Affiliated Longhua Central Hospital, Shenzhen, China

**Keywords:** Artificial intelligence, cone-beam computed tomography, deep learning, Neural networks (computer), Root fractures

## Abstract

**Objectives:**

Evaluating the diagnostic efficiency of deep learning models to diagnose vertical root fracture in vivo on cone-beam CT (CBCT) images.

**Materials and methods:**

The CBCT images of 276 teeth (138 VRF teeth and 138 non-VRF teeth) were enrolled and analyzed retrospectively. The diagnostic results of these teeth were confirmed by two chief radiologists. There were two experimental groups: auto-selection group and manual selection group. A total of 552 regions of interest of teeth were cropped in manual selection group and 1118 regions of interest of teeth were cropped in auto-selection group. Three deep learning networks (ResNet50, VGG19 and DenseNet169) were used for diagnosis (3:1 for training and testing). The diagnostic efficiencies (accuracy, sensitivity, specificity, and area under the curve (AUC)) of three networks were calculated in two experiment groups. Meanwhile, 552 teeth images in manual selection group were diagnosed by a radiologist. The diagnostic efficiencies of the three deep learning network models in two experiment groups and the radiologist were calculated.

**Results:**

In manual selection group, ResNet50 presented highest accuracy and sensitivity for diagnosing VRF teeth. The accuracy, sensitivity, specificity and AUC was 97.8%, 97.0%, 98.5%, and 0.99, the radiologist presented accuracy, sensitivity, and specificity as 95.3%, 96.4 and 94.2%. In auto-selection group, ResNet50 presented highest accuracy and sensitivity for diagnosing VRF teeth, the accuracy, sensitivity, specificity and AUC was 91.4%, 92.1%, 90.7% and 0.96.

**Conclusion:**

In manual selection group, ResNet50 presented higher diagnostic efficiency in diagnosis of in vivo VRF teeth than VGG19, DensenNet169 and radiologist with 2 years of experience. In auto-selection group, Resnet50 also presented higher diagnostic efficiency in diagnosis of in vivo VRF teeth than VGG19 and DensenNet169. This makes it a promising auxiliary diagnostic technique to screen for VRF teeth.

## Introduction

Vertical root fracture (VRF) is defined as a complete or incomplete longitudinal fracture plane that can initiate at any level of the root, usually in a buccolingual direction and is defined as one of the crack types [1–3]. VRF could occurred in both endodontically and non-endodontically treated teeth. In the Chinese population, with over 40% of the fractures occurring in non-endodontically treated teeth [4–6]. The treatment of VRF depends on a precise diagnosis and can vary from partial resection of the root to extraction [3]. However, VRF could presents subtle signs and symptoms unnoticed by the clinicians until major periapical changes occur [6, 7]. The characteristics of VRFs may lead to missed diagnosis, delay in treatment and made VRF a diagnostic dilemma in dental clinical.

The signs and symptoms are usually nonspecific for VRF in clinical [3]. Hence, distinguishing VRF from pulpal necrosis and/or periodontal disease is often challenging [3, 6]. The radiographic techniques such as periapical radiographs were developed to diagnose VRF. However, due to the overlap of adjacent structures, 2-dimensional radiographic images are limited and fracture lines are only visible when the X-ray beam is parallel to the fracture plane or when root fragments are clearly separated [8]. Recently, cone beam computed tomography (CBCT) has become widely used in dentistry and could be used in diagnosis of VRF [9]. Nevertheless, according to different authors, the diagnostic efficiency of CBCT is still lack of stability (sensitivity and specificity with 53–98% and 80–98%, respectively) because the width of fractures varies [10]. Most fractures that cause symptoms was reported with width ranging from 60 to 770 μm [11]. The voxel size of currently used CBCT system range from 75 μm to 150 μm. When the width of fractures close to the voxel size CBCT system has. The factures on CBCT images got blurred, and the diagnosis of fractures became challenging and rely on the experience of radiologist [10, 12]. A method with diagnostic efficiency like experienced radiologist for VRF diagnosis using CBCT is needed.

Deep learning (DL) is a subset of artificial intelligence (AI). The term “deep” refers to complex neural networks with multiple neural layers between the input and output layers [13]. Of these deep learning neural networks, Convolutional neural networks (CNNs) are the most widely used in medical image analysis, it could achieve the same outcome as medical professionals within a much shorter time frame [14–17]. It employs a convolutional process to learn features contained within data and could extract abundant pixel level information of images [18]. In dentistry, CNNs could be used in tooth morphological identification [18, 19], disease classification [20–22], aesthetic evaluation [23]. Attributing to the features of CNNs, they could be prospective technique for VRF diagnosis. Fukuda et al. [24] used a CNN-based deep learning model (DetectNet) to detect VRFs on panoramic radiography images. The inclusion criteria is clearly identified VRF teeth on panoramic images. However, due to the 2-dimensional imaging of panoramic radiography, the information of images CNNs could extract is limited and the diagnostic efficiency of CNN using panoramic radiography is unsatisfactory. CBCT, due to its 3-dimensional and high-resolution imaging, could provide much more detailed image information of tooth than panoramic radiography. So, CNNs based CBCT image analyzing could be adopt for VRF diagnosis. However, as far as we know, there is no research using neural network to diagnose in vivo VRF on CBCT images.

This study aimed to investigate the feasibility of CNN models in diagnosing non-endodontically treated VRF teeth on CBCT images in vivo. Moreover, an auto-tooth selection model was built before the CNN models to explore the feasibility of automatically diagnosing and screening to VRF using AI system.

## Materials and methods

### Patients and datasets

A radiology graduate student searched the picture archiving and communication system (PACS) for CBCT images between 2019 and 2021. The inclusion criteria for CBCT images of VRF teeth were as follows: (a) non-endodontically treated tooth; (b) images with good quality and without artefacts such as motion artefacts or beam harden artefacts. (c) fractures were recorded if a hypodense line was presented on at least two consecutive axial images. For CBCT images of non-VRF teeth, three types of teeth were randomly included: (a) apical periodontitis teeth caused by caries; (b) healthy teeth; (c) periodontitis teeth with horizontal bone loss. Before final inclusion, the VRF or non-VRF teeth were reevaluated and confirmed by two radiologists with more than 10 years of experience (radiologist A and radiologist B). After three months, the same radiologists reconfirm the included teeth, and intra-examiner agreement was analyzed.

In total, 216 patients (126 males and 90 females; mean age, 52.03 ± 14.29 year; range, 19 ~ 86 years) were included in the study. Of them, 138 VRF teeth were confirmed and 138 non-VRF teeth were confirmed. The approval from the Ethics Committee of the Nanjing Stomatological Hospital, Medical School of Nanjing University [2018NL-044(KS)] was obtained prior to perform this retrospective study and the requirement for written informed consent was waived by the Ethics Committee.

All CBCT images were performed using NewTom VG scanner (QR SRL, Verona, Italy) with a voxel size of 0.15 mm, 110 kV, 3.6–3.7 mA, field of view of 12 × 8 cm and acquisition time of 5.4 s.

### Image processing

#### Region of interest (ROI) selection

**Fig. 1:**
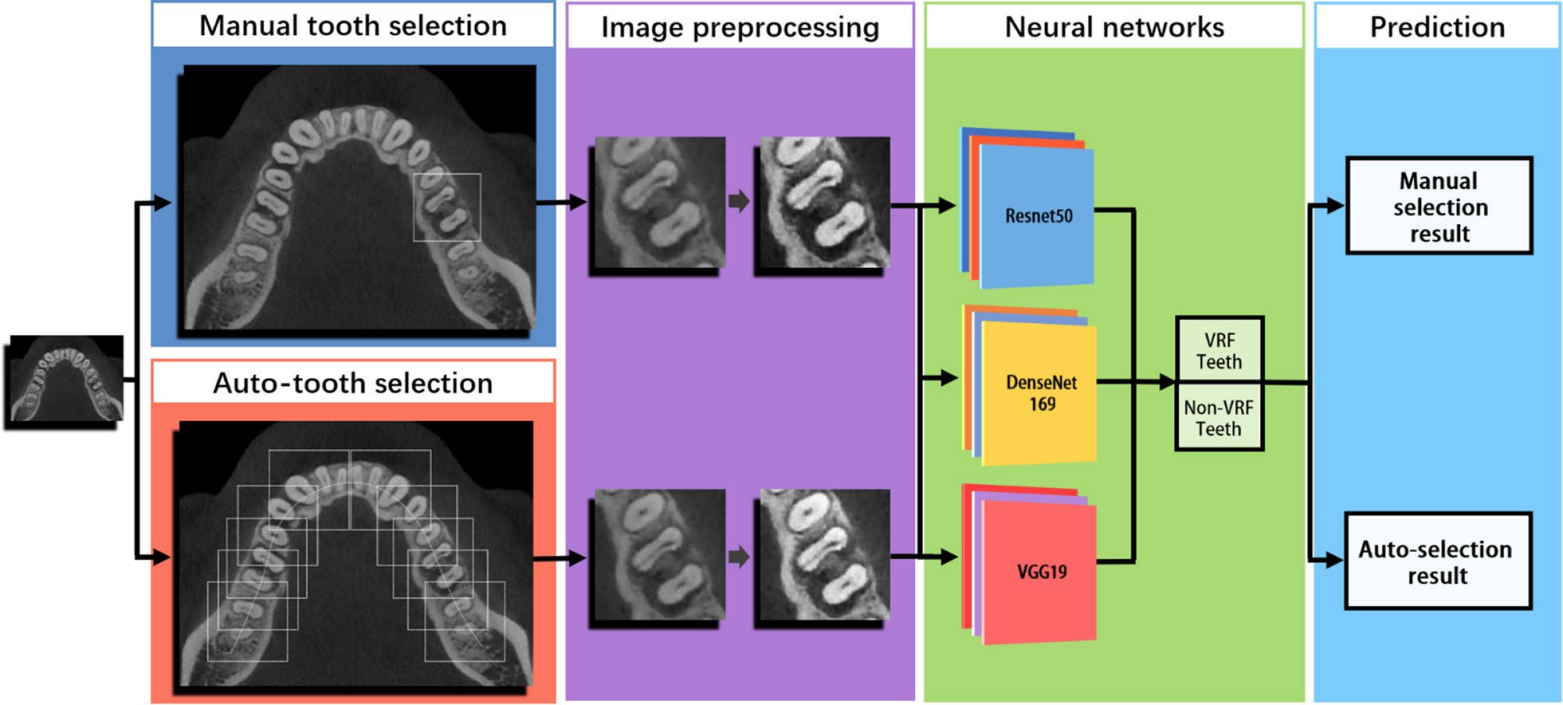
The workflow of the deep learning framework. Firstly, the same tooth on dentition images were manually selected in manual selection group and auto-selected using tooth selection model in auto-selection group. The images in two groups were then preprocessed in the same way and used as datasets to three CNN models. Finally, the three CNN models output the diagnostic result of manual selection group and auto-selection group

There were two experimental groups: auto-selection group and manual selection group. Two experimental groups used the same patients’ images. For auto-selection group, a radiologist with 5 years of experience manually cropped ROI of dentition. For each VRF and non-VRF tooth, two axial dentition images were cropped. The dentition images were used as data to tooth selection model. For manual selection group, the same radiologist manually cropped the ROI of VRF and non-VRF teeth which are confirmed by radiologist A and radiologist B using the same dentition images (Fig. 1).

#### Tooth selection model

**Fig. 2:**
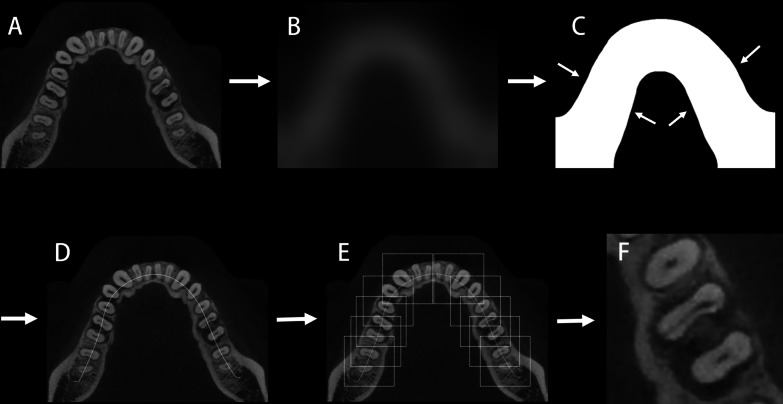
The schematic diagram of tooth selection model. **A** shows the original dentition images. **B** shows the dentition images got Gaussian blurred. The detail in image got reduced. **C** shows binary dentition images. the shape of dentition got extracted. **D** shows the moving line has been extracted and placed on the original dentition image in corresponding position. **E** shows the identification boxes has been placed on the dentition every 60–80 pixels. **F** is the cropped image original image along the outline of identification box

The tooth selection model were built through 5 steps (Fig. 2): (a) The dentition images were firstly got Gaussian blurred to reduce image detail [25]. (b) The blurred dentition images were self-adaption binaryzated to get grayscale foreground dentition images using modified Otsu algorithm [26]. The open operation and close operation were also performed to get the grayscale foreground dentition images smooth. This procedure is to select the dentition image to get the shape of foreground dentition. (c) The skeleton of grayscale foreground dentition image were extracted using K3M algorithm and the extracted skeleton were used as moving line of selection boxes [27]. (d) 170 × 170 pixels tooth selection boxes were placed on the moving line every 60–80 pixels and the tooth in selection boxes were cropped along the outline of selection boxes using algorithm (The tooth selection model was built and performed in Python 3.6).

To compare the diagnostic efficiency of manual selection group with auto-selection group. The teeth in manual selection group were identified and the corresponding teeth cropped through tooth selection model were selected as the dataset of auto-selection group. Because the selection box is larger than the manual selection ROI, a tooth may be located at two or three continue selection boxes. So, for the VRF or non-VRF tooth in each dentition image, one or two tooth images were cropped.

#### Imaging preprocessing

For manual selection group, A total of 552 cropped CBCT images (276 VRF teeth images and 276 non-VRF teeth images) were finally obtained. For auto-selection group, A total of 1118 cropped CBCT images (555 VRF teeth images and 563 non-VRF teeth images) were finally obtained.

Before putting images into CNN models, a sharpen algorithm (gray level transformation [28]) was performed to the images in both manual selection group and auto-selection group. The processed images were used as the datasets of CNN models.

### CNN models

When building the CNN models, a series of enhancements were performed on the input images to obtain more data by reducing overfitting of the model. These enhancements included random horizontal and vertical flipping, random image rotation within 90° and random brightness, contrast and saturation adjusting. After processing, the images was used as input data for the CNN models.

The CNN models were implemented on hardware with following specification: intel processor i7, 64 GB RAM with NVIDIA Tesla V100 GPU, 1 TB hard disk for implementing. Three CNN models were used to classify VRF teeth from teeth without VRFs based on CBCT images. The CNN models: VGG19, DenseNet169, ResNet50, were used as a backbone model pretrained on the ImageNet database [29–31]. A simple workflow scheme for this process is shown in Fig. 1. Three CNN models were trained in the same training sample (75% as training datasets and 25% as testing datasets). All training sessions were carried out using deep learning package Pytorch 1.11 (pytorch.org/) of Python software. The batch size was set to 16 and the learning rate to 5e-3, decreasing by a factor when no further decrease was observed in the validation dataset. We have selected the models with the best accuracy in the validation set for each algorithm. Fivefold cross-validation was used to establish the CNN models. The result was the mean of the fivefold cross-validation for the validation group. The diagnostic efficiency of manual selection group and auto-selection group were compared.

### Diagnosis of VRFs on CBCT images by radiologist

After development of the AI models were complete, A radiologist with 2 years of experience (radiologist C) manually diagnose VRFs using the same CBCT images in manual selection group. The radiologist did not take part in the model training process and was blinded to patient inclusion. Radiologist C was also unaware of patient names, clinical and imaging findings, or final diagnosis. After three months, radiologist C re-diagnose VRFs using the same CBCT images in manual selection group to analyze intra-examiner agreement.

## Statistics

The diagnostic accuracy, sensitivity, specificity and positive predictive value (PPV) of the three CNN models were calculated in manual selection group, auto-selection group and radiologist C. The receiver operating characteristic (ROC) curves and the area under the curve (AUC) of the three networks in manual selection group, auto-selection group were constructed and calculated using Pandas package (pandas.pydata.org/) of Python software. Kappa analysis was used to assess inter- and intra-examiner agreement. Statistical analysis was conducted using the SPSS 23.0 software (IBM SPSS Statistics Base Integrated Edition 23, Armonk, NY, USA).

## Result

### Diagnostic performance of manual-tooth selection group and radiologist

**Fig. 3:**
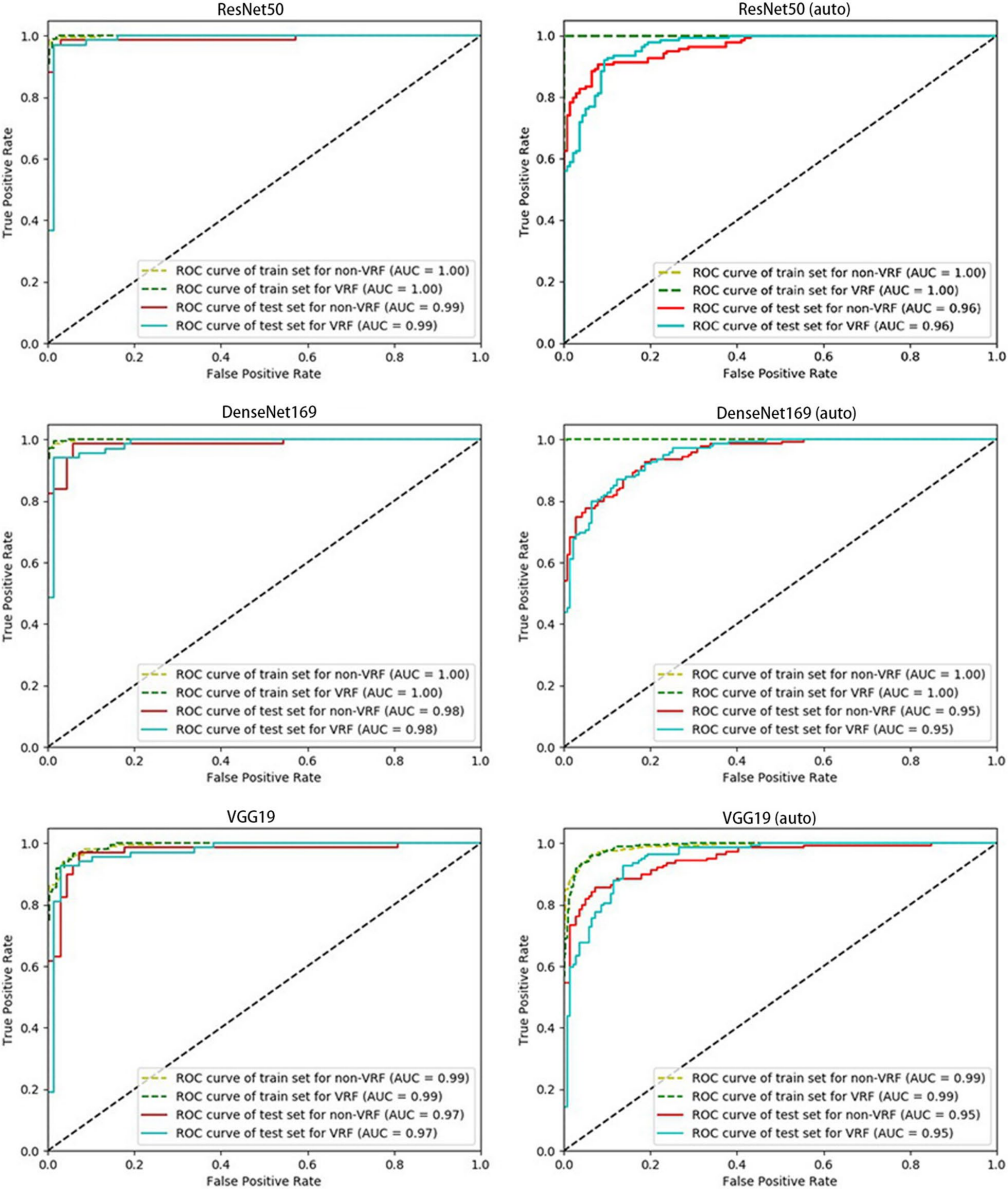
ROC curve of three CNN models in two experimental groups. ResNet50 presented the highest AUC in both manual selection group and auto-selection group with AUC of 0.99 and 0.96, respectively

**Fig. 4:**
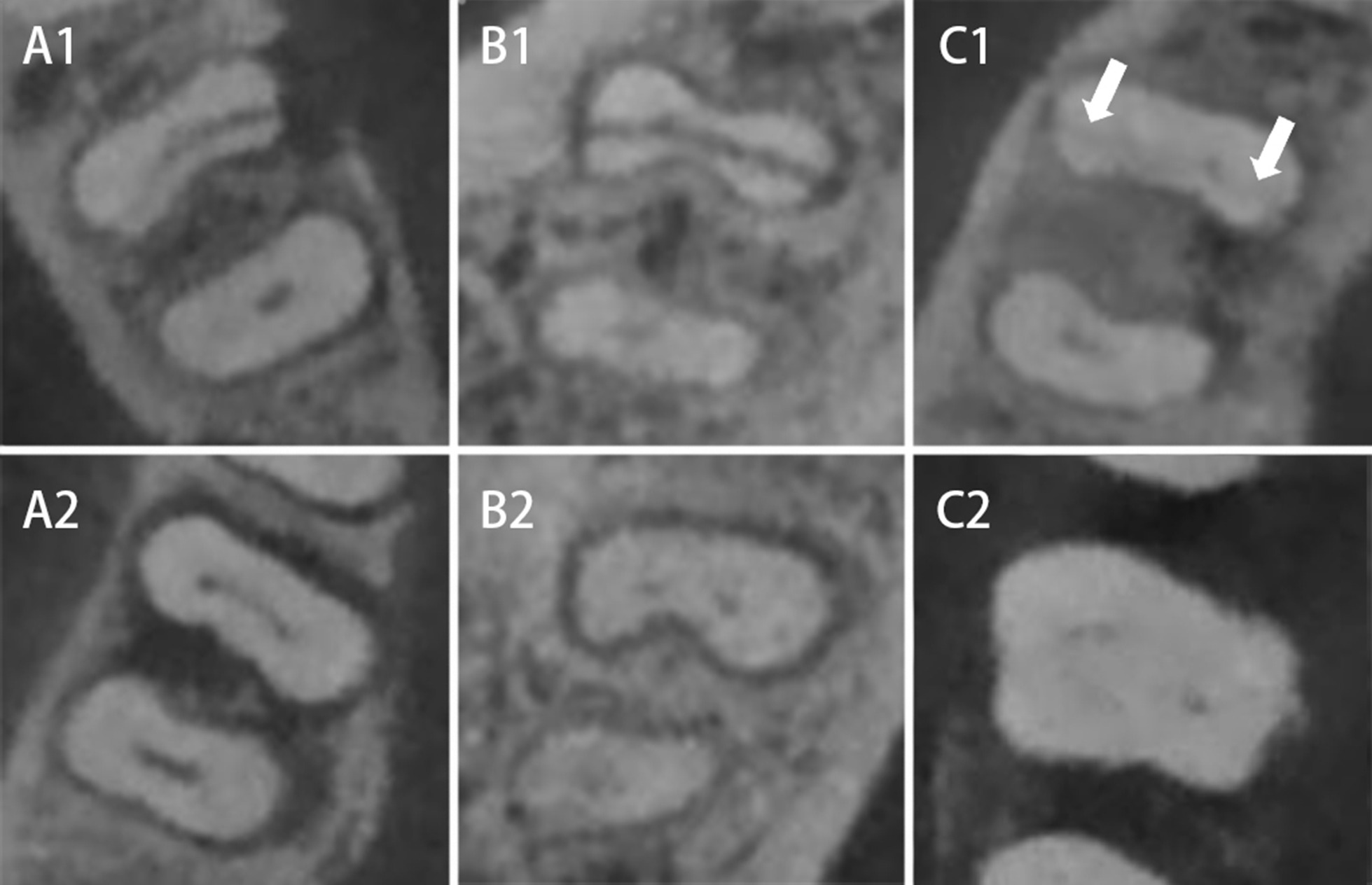
Teeth in dataset with complex symptoms. **A1**, **B1** and **C1** are VRF teeth. **A2**, **B2** and **C2** are non-VRF teeth. **A1** shows an arch low-density area (bone loss) at one side of the fracture on the CBCT image. **A2** also shows an arch low-density area (bone loss) at the lingual side of distal root on the CBCT image. However, this tooth is a non-VRF tooth. **B1** and **B2** show a low-density area around the mesial root on the CBCT image. However, **B1** is VRF tooth and B2 is non-VRF tooth. **C1** shows a subtle fracture. **C2** shows a tooth with horizontal bone loss. Low-density area is large and around the tooth. All teeth above were correctly diagnosed in manual selection group

The classification performances of three networks in manual selection group and radiologist C were shown in Table 1. The accuracy of ResNet50, VGG19 and DenseNet169 in manual selection group was 97.8%, 96.3%, 94.9% and 95.3%, respectively (Table 1). The accuracy of radiologist C was 95.3%. The ROC curves of the three networks were shown in Fig. 4. The AUC of Resnet50, VGG19 and DenseNet169 to diagnose VRF teeth were 0.99, 0.97 and 0.98, respectively (Fig. 3).

**Table 1:** The diagnostic performance of three CNN models in manual selection group and radiologist

	Accuracy (%)	Sensitivity (%)	Specificity (%)	PPV (%)
Resnet50	97.8	97.0	98.5	98.5
Densenet169	96.3	94.1	98.5	98.5
VGG19	94.9	92.7	97.0	96.9
Radiologist	95.3	96.4	94.2	94.3

### Diagnostic performance of auto-selection group

The accuracy, sensitivity and specificity, PPV of three CNN models in auto-selection group were shown in Table 2. ResNet50 had the highest diagnostic accuracy and sensitivity (91.4% and 92.1%) for diagnosing VRF teeth.

**Table 2:** The diagnostic performance of three CNN models in auto-selection group

	Accuracy (%)	Sensitivity (%)	Specificity (%)	PPV (%)
Resnet50	91.4	92.1	90.7	90.8
Densenet169	87.1	80.6	93.5	92.6
VGG19	87.8	89.2	86.3	86.7

### Inter and intra-examiner agreement

Inter and intra-examiner reproducibility (kappa value) were shown in Table 3. Radiologist A and radiologist B had almost perfect inter- and intra-examiner agreement for confirming the VRF teeth, and the radiologist C had substantial intra-examiner agreement for diagnosing the VRF teeth.

**Table 3:** Repeatability analysis of VRF teeth confirming and diagnosis

	Kappa Value	Interpretation
Inter-examiner agreement (radiologist A and B)	1	Almost perfect agreement
Intra-examiner agreement (radiologist A)	1	Almost perfect agreement
Intra-examiner agreement (radiologist B)	1	Almost perfect agreement
Intra-examiner agreement (radiologist C)	0.711	Substantial agreement

Radiologist A and B: more than 10 years of experience;

Radiologist C: 2 years of experience

## Discussion

VRFs in non-endodontically and endodontically treated teeth share common factors, such as age-related microstructural changes, the specific anatomies of the susceptible roots, biting pain, deep periodontal pockets and periodontal or periradicular radiolucency [6]. Moreover, the diagnostic result of endodontically treated teeth for radiologists will be affected by beam-hardening artefacts generated by gutta-percha. So, in this study, the VRF teeth included are non-endodontically treated.

Johari et al. [32] used feature extraction based probabilistic neural network to detect VRF on CBCT images ex vivo*.* It achieved accuracy, sensitivity and specificity as 96.6%, 93.3% and 100%, respectively. However, the root fractures in the study were artificially generated. The structure of true fractures could be more complicated [33]. Moreover, because of the motion artefacts generated when living objects breathing and heart-beating, and increased when the X-ray beam passing through a greater volume of hard and soft tissues in the body [10], the accuracy of CBCT in detecting VRFs was significantly lower compared to the ex vivo accuracy in most situation [10].

Although CBCT is a feasible radiographic technique to detect VRF, but the diagnostic efficiency of CBCT for VRF diagnosis could be unstable and affected by many factors, such as radiologist’s experience, width of fracture, CBCT system used, settings in scanning and reconstruction [9, 10, 34–39]. Of these factors, radiologist’s experience could be a crucial one. A CBCT system with 80 μm voxel size showed blurry images of VRF teeth with fracture widths of approximately 100 μm [40]. Moreover, the artefacts in in vivo CBCT scanning could also affect the diagnostic efficiency for a radiologist or clinician to diagnose VRF teeth. The radiology graduated student showed a significantly lower accuracy in diagnosis of VRF teeth than experienced radiologist [12]. In this study, the diagnostic efficiency of CNNs was compared with radiologist with 2 years of experience. Resnet50 achieved relatively higher accuracy, sensitivity and specificity than the radiologist. It could provide a stable auxiliary diagnosis tool for clinicians.

Of three CNNs models, the ResNet model could employ the entire image and is capable of retaining image information more completely than many CNN networks [31]. It exhibits high diagnostic efficiency for medical imaging analyzing [41–44]. In our study, Resnet50 achieved the best diagnostic efficiency in manual tooth selection group with accuracy, sensitivity and specificity as 97.79%, 97.06% and 98.53% respectively. Resnet 50 also showed a good stability in detection of teeth with complicated symptoms (such as non-VRF tooth with large bone loss as VRF teeth usually have, VRF tooth with less bone loss, VRF without bone loss, VRF tooth with subtle fractures [12]) (Fig. 4).

Due to nonspecific signs and symptoms, VRF tooth may got miss diagnosed [7]. Root fractures could progress to get gingival sulcus inflammatory and periodontium destructed, and may finally result in alveolar bone loss in almost all teeth [45]. An auto-diagnosis model could help clinician be aware of some non-symptom teeth. In our study, auto-selection group achieved accuracy, sensitivity and specificity as 91.37%, 92.09% and 90.65% respectively. We noticed the diagnostic efficiency of auto-selection group is lower than manual selection group. The center of auto-selection area may deviate so the tooth could not be located at the center of the cropped CBCT images. Deviated attention center could disturb CNN models analyzing and therefore cause misdiagnosis. However, this phenomenon will become less as the sample size is getting larger [46].

This study still has several limitations. Firstly, the VRF data included needs to be further expanded to get a more stable result for neural network models. Secondly, the teeth in our study are non-endodontically treated. Artefacts generated by root filling materials and metal post on CBCT images could also affect the diagnostic accuracy for VRF. The diagnostic efficiency for CNNs to endodontically treated VRF teeth on CBCT images could be explored in the future. Thirdly, auto-selection group showed a lower accuracy than manual selection group. The auto-selection algorithm may need to be further optimized. Fourthly, the VRF teeth included in our study are observable by experienced radiologist. Hidden fracture unobservable on CBCT images may need to be included for future clinical applications.

## Conclusion

In manual selection group, ResNet50 presented higher diagnostic efficiency in diagnosis of in vivo VRF teeth than VGG19, DensenNet169 and radiologist with 2 years of experience. In auto-selection group, Resnet50 also presented higher diagnostic efficiency in diagnosis of in vivo VRF teeth than VGG19 and DensenNet169. This makes it a promising auxiliary diagnostic technique to screen for VRF teeth.

## Data Availability

The [.DICOM] data and [.jpg] data used to support the findings of this study were supplied by [Zitong Lin] under license and so cannot be made freely available. Requests for access to these data should be made to [Zitong Lin, E-mail: linzitong_710@163.com].

## Abbreviations

VRF: Vertical root fracture
CBCT: Cone-beam computed tomography
CNN: Convolutional neural network
ResNet: Residual neural network
PPV: Positive predictive value
ROC: Receiver operating characteristic
AUC: Area under the curve
